# Emerging Challenges and Opportunities for Education and Research in Weed Science

**DOI:** 10.3389/fpls.2017.01537

**Published:** 2017-09-05

**Authors:** Bhagirath S. Chauhan, Amar Matloob, Gulshan Mahajan, Farhena Aslam, Singarayer K. Florentine, Prashant Jha

**Affiliations:** ^1^The Centre for Plant Science, Queensland Alliance for Agriculture and Food Innovation, The University of Queensland, Brisbane QLD, Australia; ^2^Department of Agronomy, Muhammad Nawaz Shareef University of Agriculture Multan, Pakistan; ^3^Department of Agronomy, Bahauddin Zakariya University Multan, Pakistan; ^4^Centre for Environmental Management, Faculty of Science and Technology, Federation University Australia, Ballarat VIC, Australia; ^5^Southern Agricultural Research Centre, Montana State University, Bozeman MT, United States

**Keywords:** advanced technologies, climate change, herbicide resistance, integrated weed management, research scientist, weed ecology, weed research and education

## Abstract

In modern agriculture, with more emphasis on high input systems, weed problems are likely to increase and become more complex. With heightened awareness of adverse effects of herbicide residues on human health and environment and the evolution of herbicide-resistant weed biotypes, a significant focus within weed science has now shifted to the development of eco-friendly technologies with reduced reliance on herbicides. Further, with the large-scale adoption of herbicide-resistant crops, and uncertain climatic optima under climate change, the problems for weed science have become multi-faceted. To handle these complex weed problems, a holistic line of action with multi-disciplinary approaches is required, including adjustments to technology, management practices, and legislation. Improved knowledge of weed ecology, biology, genetics, and molecular biology is essential for developing sustainable weed control practices. Additionally, judicious use of advanced technologies, such as site-specific weed management systems and decision support modeling, will play a significant role in reducing costs associated with weed control. Further, effective linkages between farmers and weed researchers will be necessary to facilitate the adoption of technological developments. To meet these challenges, priorities in research need to be determined and the education system for weed science needs to be reoriented. In respect of the latter imperative, closer collaboration between weed scientists and other disciplines can help in defining and solving the complex weed management challenges of the 21st century. This consensus will provide more versatile and diverse approaches to innovative teaching and training practices, which will be needed to prepare future weed science graduates who are capable of handling the anticipated challenges of weed science facing in contemporary agriculture. To build this capacity, mobilizing additional funding for both weed research and weed management education is essential.

## Introduction

Weeds, by virtue of their dynamic and resilient nature, represent a constant problem in agricultural production. The extent of weed infestation in the field depends on the agronomic practices used (for example, the type of crop and competitive ability of its cultivar, crop rotation, type of tillage, method and timing of fertilization, row spacing, seeding densities, and herbicides), soil type and fertility status, and prevailing environmental conditions (Chauhan et al., 2012; Swanton et al., 2015). Being a botanical pest, weeds share the same trophic level as crop plants, and weed-crop competition for light, water, and nutrients results in substantial crop yield losses (Swanton et al., 2015; Ramesh et al., 2017). A successful weed management program tends to integrate two objectives simultaneously: (i) prevent yield loss owing to weed competition in the short term, and (ii) avoid the addition of weed seed/vegetative propagules to the soil seed bank, to reduce weed densities in subsequent years (Battle et al., 1996). The advent of diverse herbicide molecules for selective weed management has revolutionized contemporary agriculture, which has become more productivity-oriented than ever (Shaw, 1964; Hamill et al., 2004).

Herbicide use for weed control in agricultural crops has made agricultural production simpler and economical (Johnson et al., 2009), resulting in increased farm size. On the other hand, with the increased availability of selective herbicides for weed control, ecologically sustainable weed management as an integral component of cropping systems seems neglected. Although herbicide-based agricultural systems have benefited the farming community in many ways, continuous use and heavy reliance on herbicides has resulted in recurrent evolution of herbicide-resistant weeds, shifts in the spectrum of weed flora, and contamination of the surrounding environment, mainly through water movement (Duary, 2008; Johnson et al., 2009). Consequently, there is an ever-growing consensus that the design of weed management systems with reduced reliance on herbicides seems essential to overcome the ill-effects associated with over-reliance on herbicide usage (Bastiaans et al., 2000; Hatcher and Melander, 2003; O’Donovan et al., 2007). Therefore, the challenge faced by weed scientists is to develop innovative, ecologically sound, economical, and sustainable weed management systems, which can be integrated into existing and future cropping systems to bring a more diverse approach to weed management. Due to the genetic diversity and developmental plasticity of weed communities, weed management programs are now considered a continuous process within agricultural systems. New challenges, like herbicide-resistant biotypes, invasive plant species, and climate change have compelled weed researchers to develop cutting-edge technologies. The dynamic nature of weeds will continue to pose multi-dimensional problems for research scientists, and the quest to find innovative solutions to these challenges may once again revolutionize agriculture.

An additional confounding factor in this essential activity is that the goals and directions of weed science, which were clearly defined and universally recognized in the past, appear to have lost clarity in recent times (Breen and Ogasawara, 2011). Several researchers have speculated whether weed science is moving in the right direction, and as a result questioned whether it has been able to make practical impacts on current emergent problems (Wyse, 1992; Coble, 1994; Hall et al., 2000; Fernandez-Quintanilla et al., 2008; Moss, 2008; Breen and Ogasawara, 2011). Indeed many authors have specifically commented that the contribution of research into weed biology and ecology toward sustainable weed management programs is not up to the mark, and will require more systematic and focused work (Mortensen et al., 2000; Chauhan and Johnson, 2010). Similarly, a later study suggested that, even in the herbicide era, knowledge of weed biology seems indispensable and could serve as the basis for practical weed management (Van Acker, 2009). Diverse approaches that can relate weed biology studies to practical weed management are needed in this regard. Cousens (1999) argued that although weed threshold levels form an important area of research in weed science, these were seldom exploited practically. The author criticized the multitude of phenomenological experiments and over-dependence on simulating repetitive case studies, which have actually transformed weed science discipline into weed technology. Echoing the urging of Wyse (1992), the study emphasized the need for a greater understanding of the basic principles underpinning weed science, besides a paradigm shift regarding critical research questions, changing from documenting “what occurs” to “why things happen.” Ward et al. (2014) in their critique of agricultural weed research, identified two major aspects for improvement of the weed science discipline: (i) scientific studies must be reoriented toward an understanding of weed biology, and (ii) management efforts to minimize the negative impact of herbicides. These authors criticized weed research conducted in recent years as being characterized by a high degree of repetitiveness, with an excess of purely descriptive studies that fail to relate novel hypothesis with established ecological and evolutionary facts. The authors urged the need to revisit agricultural weed research in a more holistic manner, comprising of a broader vision, a deeper theoretical justification, and an inter-disciplinary approach.

It has been suggested that the domain of crop protection, which intimately includes studies in weed science, needs to switch from technology-oriented to system-oriented tactics, which acknowledge innovation as a perfect blend of technological and non-technological (institutional and social) advancements across various levels, stretching from the field to the farm and the region (Schut et al., 2012). In this respect, a rigorous introspective analysis of deliberated goals, resources in hand, directions, progress evaluation, and dissemination of results to the intended audience are key considerations for weed science in the foreseeable future, so that the discipline emerges stronger and more focused. Here, in a positive and constructive manner, we intend to highlight and prioritize the current issues for weed science research and education, identify challenges and opportunities, and critically assess what can be done to push the frontiers of weed science research and embrace horizons of quality-oriented weed science education. We would like to emphasize that the information presented in this article is deliberately general and not specific tailored to a particular climate or country.

## Emerging Issues in Weed Science

### Herbicide Resistance and Weed Plasticity

Over-reliance on herbicides as the sole tool to control weeds, and continuous use of herbicides with similar modes of action (MOA), have led to the evolution of herbicide-resistant weeds. Multiple herbicide-resistant species like the *Amaranthus* complex in corn and soybean, and grass weeds (*Echinochloa* spp., species of *Aegilops*, *Alopecurus*, and *Lolium*, *Phalaris minor* Retz., etc.) in cereals and cereal-based rotations (*Avena fatua*, *Chloris truncata*), have seriously limited available herbicide options (Beckie and Tardif, 2012; Vencill et al., 2012; Heap, 2017). The evolution of resistance to glyphosate in *Sorghum halepense*, and the dispersal of resistant biotypes both by seeds and rhizomes, first in Argentina and then in the United States (Heap, 2017), is another significant example. In fact, 270 herbicides covering the global market represent only 17 MOA, and almost half of them act as acetolactate synthase (ALS), photosystem (PS) II, and Protox inhibitors (Macías et al., 2007). The paucity of new/novel herbicide MOA discovery in the last 20 years has deterred weed control and prompted herbicide resistance (Macías et al., 2007; Beckie and Tardif, 2012; Duke, 2012).

The widespread adoption of glyphosate-resistant crops and the use of a single herbicide (glyphosate) for weed control since the mid-1990s has diminished the quest for a new herbicide MOA (Green, 2011; Beckie and Hall, 2014; Heap, 2014; Duke, 2015; Owen et al., 2015). The number of active ingredients used in at least 10% of the soybean area in the United States has dramatically declined from 11 in 1995 to just 1 in 2002 (Green, 2011). Regulatory authorities sometimes deregister old herbicides for unscientific reasons; thereby limiting the diversity of chemicals available for weed control and increasing reliance on fewer active ingredients, leading to increased selection pressure (Gressel, 2011). Most of the time the de-registration is due to these two reasons: (i) lack of efficacy due to increased resistance by the target weed species; and (ii) negative effects on the environment, due to excessive persistence, leaching properties or endocrine disruption in animal species. General weed problems in speciality crops/vegetables are on the increase due to the disappearance of old herbicides and the lack of new herbicide molecules.

High plasticity in weeds facilitates season-long germination in many species (Zhou et al., 2005). With extended exposure to a given situation, plasticity enables weeds to adapt to a wide range of environmental conditions, resource constraints and intervention practices. Genetic diversity is the reason underpinning plasticity in weeds. Norris (1992) conducted isozyme analysis of several weed species, and concluded that even the same weed species collected from different areas showed variability at enzymatic levels. Similarly, Renton (2013) suggested that the evolution of resistance to herbicides is often modeled at a population level, but population-based methods ignore important aspects of variability between individuals within populations that may be essential drivers of resistance. Therefore, the research areas of genetics and evolution of weeds need to be strengthened. More in-depth studies are needed to develop an understanding of the various mechanisms of weed adaptation in response to changes in resources. There is much to learn about morphological, physiological, and genetic plasticity in weeds in response to the maternal environment, and understanding the effect of such plasticity on inter- and intra-specific competition could be useful for designing effective integrated weed management (IWM) programs (Bajwa et al., 2015; Mahajan et al., 2015). Weeds respond to a change in agricultural practices. For instance, an increase in surface-germinating weeds (small-seeded dicots and grasses) due to increased adoption of conservation tillage (e.g., no-till) has been observed (Price et al., 2011; Chauhan et al., 2012). The evolution of herbicide resistance in weed biotypes is another classical example of weed plasticity, which again emphasizes the need to understand the evolutionary dynamics of resistance development in weed species in order to develop effective mitigation programs (Neve et al., 2009).

### Gene Flow from Herbicide-Resistant Crops

Crop-related weed species are already an issue in herbicide-resistant crops in countries such as United States which adopted these crops a long time ago (Green, 2011; Duke, 2015). However, these species are an emerging issues in countries, which are now adopting herbicide-resistant crops, for example, Malaysia. Some examples of these species are *Oryza sativa* f. *spontanea* (weedy/red rice) in direct-seeded rice, *Aegilops cylindrical* and *Elytrigia repens* in wheat, cruciferous weeds in rapeseed, *Helianthus annuus* in sunflower crop, *Sorghum halepense* and *Sorghum bicolor* (shatter cane) in sorghum. Worldwide, weedy rice has now become a major issue in rice production systems. The introduction of imidazolinone-tolerant rice has caused a huge infestation of weedy rice because of evolution of imidazolinone-resistant weedy rice (Kraehmer et al., 2016).

The potential for gene flow from herbicide-resistant crops to wild/weedy relatives via pollen is a major concern. For example, weedy rice in the United States has evolved resistance to herbicides used in herbicide-resistant rice. The probability of gene flow may increase further if herbicide-resistant volunteer crops are followed in rotation with cross-pollinated crops, for example, corn with soybeans and oilseed rape/canola with sugar beets (Beckie and Owen, 2007). The number of scientific papers disapproving the risks of gene flow from transgenic crops to feral weedy relatives far exceeds than those explaining “how to deal with this issue.”

### Misconceptions about Integrated Weed Management and Neglected Areas of Research in Weed Science

There are misconceptions about the concept of IWM and the approach has not been followed in its true essence (Harker and O’Donovan, 2013). The execution of real IWM programs demands more efficient and diverse approaches, rather than just relying on herbicides (e.g., sequential application and tank mixtures). To date, weed research is more oriented toward herbicide research and more funding is released in this direction. Several critics have argued that weed science is a “science of herbicides” rather than the “science of weeds” (Wyse, 1992; Harker and O’Donovan, 2013). The authors examined weed science publications from 1995 to 2012, and found that more publications had been produced about chemical control rather than an integrated approach (Harker and O’Donovan, 2013). Country wise, the United States had the highest publications in weed science. When related to population size, Switzerland, the Netherlands, New Zealand, Australia, and Canada had produced a disproportionately high number of articles on IWM. In IWM, the emphasis is on diversity of weed control methods rather than relying on one single method of weed control. Therefore, in its true sense, IWM means reducing the selection pressure for development of resistance to any single method of weed control.

Cultural manipulations (tillage, sowing time, planting pattern, cover crops, row spacing, fertilizer, and water management) in IWM may complement and substitute for herbicides by contributing “many little hammers” on weeds (Liebman and Gallandt, 1997). Successful IWM tactics require advanced knowledge of weed ecology and biology (Liebman et al., 2001). Weed biology and ecology (understanding of weed species and the role they play in agro-ecosystems) remained an orphan until recently, especially in developing countries, as it was overshadowed by the success of chemical weed control (Gressel, 2011). Weed seed dormancy is an important consideration for IWM programs, which has implications for seed bank dynamics and periodicity (Chauhan and Johnson, 2010), yet its prediction remains a challenging task due to the complex nature of functional relationships between biological processes and environmental variables. This issue has affected the overall prediction of extent and timing of weed emergence in agricultural systems. Increased seed dormancy and delayed germination have often been related to herbicide resistance (Owen et al., 2015; Kumar and Jha, 2017). In a recent study, the glyphosate resistance and temperature mediated seed dormancy in some glyphosate-resistant populations was reported to reflect co-selection of resistance and avoidance imposed by decades of intensive cropping practices (Kumar and Jha, 2017). True IWM options in oilseeds and pulses are very limited (Sardana et al., 2017). For these crops, IWM with hand weeding was suggested in most of the research articles.

It has also been observed that early career weed scientists focus much of their research on herbicide efficacy, considering it a relatively easy field to publish their research, while IWM research requires more time and innovative ideas for publication. So in this way, IWM research is neglected. Considering the importance of IWM research, if at least one special issue on IWM research after 2–3 years is published in reputed journals, this could be an important step in promoting IWM research. A voluminous body of knowledge is available on the interaction of herbicides with other cultural practices. Nevertheless, the paucity of papers that evaluate economics, off-target damage, public health, education and training, ethical issues, and policy perspectives suggests that these issues are not considered priorities (Hall et al., 2000; Davis et al., 2009).

Most of the publications dealing with weed biology and population dynamics are descriptive rather than explanatory. In order to have a better understanding of the subject matter, studies must unravel why weeds respond the way they do.

### Herbicide Related Contamination

There is growing concern about herbicide residues in crop produce, soil and contamination of ground water. Besides contaminating soil and water, herbicides are known to interfere with soil enzymatic and microbial functions, which are essential for many reactions and transformations regulating soil health (Hussain et al., 2009). Recently, the impact of herbicide application on soil function was reviewed (Rose et al., 2016). The authors suggested that herbicide application could significantly alter soil function, for example, disruptions to earthworm ecology in soils exposed to glyphosate and atrazine and site-specific increases in disease resulting from the application of a variety of herbicides. The authors also suggested that sulfonylurea herbicides could affect N-fixation, mineralization, and nitrification at recommended or slightly higher application rates.

### Lack of Trained Weed Scientists in Developing Countries

Unlike entomology and plant pathology, there are no or very few departments devoted solely to weed science in agricultural universities. Moreover, few if any university faculties are assigned to weed control in fruit orchards, ornamentals, aquatic, forest, and pasture. The number of faculties devoted to non-cropland weed management, turf, vegetables, ecology, and statistical issues pertaining to weed science is also limited (Derr and Rana, 2011). Moreover, different degree-awarding institutes from various locations within a country, especially in developing countries, have almost homogenous weed science curricula, that do not consider regional variations in weed species, management practices, crops and cropping sequences, input levels, socio-economic backgrounds, and weed management skills of farmers.

### Climate Change

Lack of precise information on the effect of climate change on agricultural pests, particularly weeds, remains a major impediment to portraying a true picture of this issue (Ramesh et al., 2017). Nevertheless, the substantial environmental, ecological impacts and economic costs warrant the need to unravel these interactions on a priority basis (Ziska and McConnell, 2015). Studies dealing with the effect of CO_2_ have considered weeds and crop species as separate entities; nevertheless, under natural settings, weeds grow simultaneously with crops. Growing species in isolation to predict competitive effects as a function of elevated CO_2_ may lead to inadequate quantification of crop-weed competition, since it is very rare to see a field infested with a single weed species (Ziska and Goins, 2006). A limited number of studies have quantified the response of crops and weeds to CO_2_ in competitive environments (Ziska, 2004; Ziska and Goins, 2006), and there is an urgent need to conduct further research with mixtures of weeds and crops. Further, the impact of elevated CO_2_ on the geographical distribution of weeds in managed ecosystems also needs attention (McDonald et al., 2009). Studies of changes in floristic composition of weed communities in response to crop establishment methods, alternate moisture and tillage regimes, and other cultural practices are copious in the literature. However, studies focusing exclusively on changes in weed communities against the backdrop of elevated CO_2_ are scant (Koizumi et al., 2004), despite the probability that this could affect the overall structure and function of crop field ecosystems. Weeds once considered minor pests could became problematic due to a shift in their range caused by climate change (Peters et al., 2014).

There are strong indications that herbicide efficacy is decreased at higher CO_2_ concentrations (Ziska and Teasdale, 2000; Ziska et al., 2004), due to CO_2_-induced morpho-physiological and anatomical changes in plants that interfere with uptake and translocation of herbicides (Ziska and Teasdale, 2000; Manea et al., 2011). This implies an overall decrease in herbicide efficacy due to the dilution effect, rendering available herbicide options less effective and requiring more herbicide input to achieve the same level of weed control. Ziska et al. (2004) suggested that a greater root to shoot ratio and subsequent below ground dilution of glyphosate increased glyphosate tolerance at elevated CO_2_. Under such a scenario, perennial weeds are expected to become more problematic and difficult to control with glyphosate (Ziska and Goins, 2006). Ziska et al. (2011) postulated that a rise in atmospheric CO_2_ concentrations can have a profound effect on the biological processes of weed species, adding to their invasion potential. For example, these authors reported a 70% increase in the growth of *Cirsium arvense*, an invasive perennial C_3_ weed species. Lee (2011) showed that increased temperature had a more significant effect on plant phenological development than elevated CO_2_. Increased temperature is expected to offset the benefits of increased CO_2_ by limiting the reproductive output. If so, weed community dynamics and crop-weed interactions will need to be reassessed. There is no consensus over future rainfall prediction, except that it will become erratic, and consequently floods and droughts would become recurrent phenomena. Prolonged drought spells will favor C_4_ and parasitic weeds like *Striga hermonthica*. In contrast, under increased moisture availability, weeds like *Rhamphicarpa fistulosa* would thrive (Matloob et al., 2015). In rice, switching to direct seeding from transplanting in the quest of water saving has already increased weed competition and altered weed dynamics (Matloob et al., 2015). Frequent rain showers will limit the “rain safe periods” available for herbicide application, besides promoting leaching of soil-applied herbicides and triggering subsequent ground water contamination (Ramesh et al., 2017). Much of the research on climate change has focused entirely on manipulating the plant response to CO_2_ concentration, while neglecting the rise in temperature and drought (Bunce and Ziska, 2000; Fuhrer, 2003).

Climate change has led to altered distribution of weeds, for example, the appearance of *Marsilea* spp. under wetter conditions in rice in India. Water scarcity is driving the switch to direct-seeded rice, promoting recalcitrant grass weeds like *Dactyloctenium aegyptium*, *Eleusine indica*, *Leptochloa chinensis*, and weedy rice (*O. sativa*) in aerobic rice (Chauhan et al., 2014; Matloob et al., 2015). In the wake of climate change, variations in temperature have caused shifts in weed flora. For example, *Ischaemum rugosum* Salisb. was commonly seen in the tropical part of India, but has now become very common in the northern part of India (Mahajan et al., 2012). Increasing problems of parasitic weeds (e.g., *Striga* spp.*, Orobanche* spp.) under continuous cultivation of host crops (e.g., corn, sorghum, rice, sunflower, legumes, and vegetables), combined with low soil fertility particularly in the tropical countries, are observed. These weeds are expected to extend their geographic range under predicted climate change, affecting productivity of rainfed corn, sorghum, and rice crops. Unluckily, very few selective herbicides or other control options are available for their control, which is very difficult and is one of the obvious reasons why research has not been prompted in this regard (Zimdahl, 2007). For instance, at present the use of imidazolinone-resistant corn (mutants with herbicide seed coating) is the only known herbicide mechanism to control the parasitic *Striga* (Kanampiu et al., 2009). Infestation of wheat fields by *Phalaris minor* is expected to worsen with an anticipated rise in CO_2_ (Mahajan et al., 2012). Similarly, weedy rice will be more problematic in cultivated rice fields (Ziska et al., 2010). In crux, this reflects the potential of increased weed pressure and subsequent competition in the rice-wheat cropping system of the Indo-Gangetic Plains.

Except for several short-term bioassay studies, research efforts to envisage weed biology against the backdrop of climate change continue at a slow pace, especially with regards to long-term, system-level experiments. Such research is not only complex and long-term, but also requires an inter-disciplinary approach. Hence, it seems less attractive to funding agencies, and is likewise, unappealing to weed scientists. Invasive plant species continue to expand in number and geographic range, and are an escalating threat to managed and natural ecosystems. Against the backdrop of climate change, invasion by alien plant species has emerged as the greatest challenge to ecosystem function and stability (Hellmann et al., 2008). Derr and Rana (2011) pointed out a shortage of weed science faculties and dedicated courses aiming to improve invasive plant management, even in a technologically advanced country like the United States.

## Opportunities

### New Avenues of Weed Science Research

To understand the changes in geographic distribution of weed species, weed surveying and mapping techniques need to be updated. Weed prediction maps and decision-making tools should be developed for each particular region. Knowledge of drones [unmanned aerial vehicles (UAVs)] should be imparted to weed scientists and crop consultants so that they can use this technology in developing decision-making tools (Lopez-Granados, 2011). Remote sensing technologies offer oppurtunities to develop timely and accurate scouting and prescription maps to improve weed management decisions and protect the environment by applying more site-specific control measures (hand-weeding, targeted tillage, or spot spray) (Shaw, 2005). The use of advanced optical-sensor based sprayer technology for site-specific herbicide applications is still in its infancy stage. Similarly, hyperspectral imaging to differentiate between crop and weed biotypes is a relatively new concept (Okamoto et al., 2007), and would be a step forward in achieving precision weed control goals to reduce reliance on herbicides by applying them only where they are needed, compared with the current practice of broadcast herbicide applications. More research is needed on other precision herbicide application technologies, such as the use of shielded sprayers or herbicide banding, to reduce herbicide load and minimize herbicide runoff in furrow-irrigated cropping systems (Davis and Pradolin, 2016). The potential utility of nanoherbicides and field robots for precise weed control can also be explored. For example, an Australian university has developed a fully-autonomous weed-killing robot (AG-BOT) and claimed that it would help in cutting the cost of weed control by 90% and potentially save the farm sector $1.3 billion a year (Anonymous, 2016). Its abilities range from scouting, to knocking out weeds, to spot spraying, to the precision application of chemicals and fertilizers (Pinter et al., 2003).

Nanotechnology can play a pivotal role in achieving more efficient and targeted herbicide application (Pérez-de-Luque and Rubiales, 2009). Nanoherbicides as a “smart delivery system” provides an eco-friendly approach through reducing herbicide inputs, as well as providing control over where and when an active ingredient is released (Pérez-de-Luque and Rubiales, 2009). Herbicide formulations with particle size ranging between 100 and 250 nm manifest higher solubility in spray mixture and absorption by plants (Parisi et al., 2015). Additionally, a liposome-based biosensor has been reported to facilitate pesticide detection, which has implications for monitoring plant health and environmental conditions (Vamvakaki and Chaniotakis, 2007). However, the advantages, risks and economic viability related to robotics and nanotechnology need to be assessed. There may be some environmental health risks posed by air-borne nanoparticles. They may impair translocation of water, nutrients, and photosynthates in plants by entering through vascular tissues. The nanoparticles may enter into the lung and blood stream of humans and cause inflammation, protein fibrillation, and induce genotoxicity (Hoet et al., 2004). These risks could be avoided if the nanoparticle herbicides are injected into the soil.

The extraordinary seed production potential of annual weeds, coupled with the establishment of persistent seed banks, warrants the need for strategies that can avert seed input rather than merely focusing on reducing weed density to minimize crop yield losses (Norris, 1999). In this direction, harvest weed seed control (HWSC) methods aimed at targeting weed seed production and their return to the soil weed seed bank can have a long-term impact on weed population dynamics under field conditions (Shaner and Beckie, 2013). The concept is gaining popularity, and a non-chemical weed management tool named the “Harrington Seed Destructor” has been successfully implemented in Australia (Walsh et al., 2012). Besides this tool, chaff carts and narrow windrow burning treatments have been shown to reduce *Lolium rigidum* emergence by 55% relative to the untreated plots (Aves and Walsh, 2013).

Advances in molecular biology and biotechnology has revolutionized agriculture by enabling the development and commercialization of herbicide-resistant crops, which have been developed using both transgenic (integration of transgene) and non-transgenic (traditional plant breeding or mutagenesis) approaches (Green, 2012). Herbicide-resistant crops, particularly glyphosate-resistant (Roundup Ready) crops, have been widely adopted by growers as they offered simple, effective and economical solutions for managing a broad spectrum of weeds, with improved crop yields, less inputs, and higher net returns (Powles, 2008; Green, 2012). Advanced knowledge of the mechanisms, spread, and stability of herbicide-resistant weeds has helped in opening a new door for managing herbicide-resistant weeds in future. For example, three lessons from glyphosate resistance mechanisms, viz. target-site mutation, gene amplification, and altered translocation due to rapid vacuole sequestration, were learnt from glyphosate-resistant weeds (Sammons and Gaines, 2014). The diversity of these types of mechanisms advanced our knowledge of plant physiology and molecular biology in the past 30 years, and yet “the agricultural chemical industry has not brought any new herbicides with novel sites of action to market in over 30 years, making growers reliant on using existing herbicides in new ways” (Heap, 2014). The rapid evolution of glyphosate-resistant biotypes indicates that no herbicide is invulnerable to resistance (Powles, 2008). The rapid evolution of glyphosate-resistant weeds (37 weed species worldwide) (Heap, 2017) prompted the development of new herbicide-resistant, stacked-trait crops, in combination with the glyphosate-resistant trait (Reddy and Jha, 2016). These include glyphosate-glufosinate in soybean, corn and cotton; glyphosate-ALS inhibitors in soybean, corn and canola; glyphosate-glufosinate-2,4-D in soybean and cotton; glyphosate-glufosinate-dicamba in soybean, corn, and cotton; glyphosate-glufosinate-HPPD inhibitors in soybean and cotton; glyphosate-glufosinate-2,4-D-ACCase inhibitors in corn; and glufosinate-dicamba in wheat (Green, 2014). However, these stacked-trait crops will not be an ultimate weed management solution because several weeds have already evolved resistance to these herbicides, and an effective stewardship program is a must (Reddy and Jha, 2016; Heap, 2017). Beckie and Hall (2014) stated that stacked herbicide-resistant (HR) traits (e.g., glyphosate+glufosinate+dicamba) would provide a short-term respite from HR weeds, but will perpetuate the chemical treadmill and selection of multiple-HR weeds. The only sustainable solution is for government or end-users of commodities to set herbicide-use reduction targets in major field crops similar to European Union member states, and include financial incentives or penalties in agricultural programs to support this policy.

Molecular biology tools have been utilized to understand the genetics of herbicide resistance evolution in weeds, but their practical implications to develop long-term approaches for herbicide-resistance mitigation in diverse agroecosystems are still limited. Molecular-based approaches for diagnosis of herbicide resistance in weeds are more efficient and less labor intensive than the traditional methods of conducting whole-plant herbicide dose-response bioassays (Corbett and Tardif, 2006). DNA-based molecular markers provide a tremendous opportunity to study weed genetic diversity and hybridization among related weed species. Simple sequence repeats (SSRs), microsatellites, amplified fragment length polymorphisms (ALFPs), and inter-simple sequence repeats (ISSRs) have more recently been used in weed genomic studies (Horvath, 2010). For instance, molecular markers were used to study the hybridization and transfer of herbicide-resistance trait between *Amaranthus palmeri* and *A. tuberculatus* and between *A. tuberculatus* and *A. hybridus* (Trucco et al., 2005a,b). PCR-based markers can be used to study weedy characteristics such as dormancy, seed shattering, and biotic and abiotic stress tolerance (Horvath, 2010).

The novel “Omics” technology can revolutionize weed science. Genomics, proteomics, transcriptomics, and metabolomics are perceived as the beginning of a potential new era for the management of resistant weeds (Neve et al., 2014) The advent of biochemical and molecular techniques, in conjunction with computational tools, has made it possible to incorporate protein modeling and crystallography to unravel target site mutations (Tranel and Horvath, 2009; Horvath, 2010; Shaner and Beckie, 2013). This is a potential tool to screen for possible resistance before a herbicide is marketed (Hollomon, 2012). Global gene-expression profiling techniques, such as microarrays, have been suggested as an effective tool in studying non-target site mechanisms (Yuan et al., 2007). *De novo* whole genome sequencing, specifically EST (expressed sequence tag), can be used to identify various ‘candidate genes’ involved in mediating specific physiological and biochemical processes in a weed. These tools will also provide valuable insights into the response of weeds to biotic and abiotic stresses and crop-weed competition (Tranel and Horvath, 2009; Horvath, 2010); hence, improving our understanding of the invasiveness of weeds, which would ultimately allow development of long-term, IWM strategies. Recently, a small subgroup of weed scientists has entered into the genomic era (Gressel, 2011). There is a need to train young weed scientists in developing as well as developed countries in the field of molecular biology for a better understanding of crop and weed resistant traits.

The RNAi technology branded as BioDirect^TM^ by Monsanto, although at an infant stage (Hollomon, 2012), has produced a mirror copy of weed DNA in which target genes can be turned on and off. The exploitation of precise RNA segments capable of inhibiting enolpyruvyl-shikimate-3-phosphate synthase (EPSP) proteins in plants is a major breakthrough in reversing resistance (Shaner and Beckie, 2013); however, this mechanism only applied to glyphosate.

The quest to identify the compounds that can induce weed seed germination on demand has been fulfilled by the discovery of novel compounds like karrikinolide (Long et al., 2011). Karrikinolide is a biologically active component of smoke and a strong stimulant of weed seed germination (Daws et al., 2007; Stevens et al., 2007). The practical application of this compound is to synchronize weed seedling emergence by stimulating germination, hence, it is a potential tool to deplete weed seed banks.

### Weed Science Education

At the start of this reflection on weed science education, we see that students enrolled in weed research should be encouraged to conduct their trials in farmers’ fields, and so develop their mindset toward practical research. There is a need to ascertain why the intake of students in the weed science discipline is declining day by day, while on the other hand, problems and issues related to weed science are increasing. Identifying the causes for this decline in student intake, and finding workable solutions, is important for weed science. There are therefore two imperatives here (i) the area of weed science and weed management needs to be presented to the community in a more positive light, in order that potential students will be attracted to the discipline, and (ii) there is a need to develop interdisciplinary programs in weed science, to allow students to learn more about the complexity of weeds in farming systems, and eventually discover and implement new solutions (Davis et al., 2009; Mortensen et al., 2012). New curricula in weed science should be focused on the role of genetics, evolutionary biology, molecular biology, and biochemistry.

We agree that degree programs in weed science with more diverse curricula, that emphasize locally important weed issues, and provide practical training in laboratory and field-based skills, will foster critical thinking and an ability to tackle complex weed situations. Additionally, it would be a good step forward if graduates from weed science could have more hands-on experience, funded through industry scholarships (Davis et al., 2009). Such changes to weed science education will ensure the availability of technically trained manpower personnel (weed science professionals) to cope with future challenges in weed management.

### Implementation of Need-Based Weed Research

We assert here that there is a need to develop weed management programs based on knowledge of weed ecology in relation to maternal environment, genetic and biochemical aspects, and molecular biology. In designing an appropriate weed science curriculum, the economics related to both weed-induced yield loss and weed control methods need to be incorporated. If farmers are not convinced of the economic feasibility of new weed control methods, then even sound and scientifically strong innovations may not be adopted. There is a need to identify weed threshold levels in important crops as part of precision weed control, so that weed control practices can become sustainable even with a reduced load of herbicides (Norris, 1999).

Precision weed control systems may optimize the use of herbicides by allowing site-specific weed management and weed seed prevention from survivors, achieving zero seed thresholds, and therefore, may aid in mitigating herbicide-resistant weeds (Reddy et al., 2014). Modeling studies on crop-weed competition need to be explored for making long-term weed control strategies. It is inevitable that long-term studies are needed in weed science, especially in weed ecology, weed resistance evolution, and herbicide-resistant crops. Therefore, private companies should come forward with funding for students’ and young scientists’ projects. We have identified that diversified weed control tactics are needed for sustainable weed control. IWM, including cover crops, tillage, row spacing, and crop density, needs to be explored for long-term weed control in different crops to minimize use of herbicides. At the same time, research in weed science must be oriented toward farmers’ needs and reflect their feedback in order to provide economical solutions to weed infestation, while protecting future generations through an insistence on truly and sustainable weed mitigation strategies. At the pragmatic level, farmers’ participation research regarding weed science needs to be strengthened in order to ensure the development of practical and sound decision-making tools (Hall et al., 2000).

## Future Directions

As a contribution to this debate, we offer here a 14 point discussion list, which we think should form the basis of a serious re-examination of the current approaches to the direction of research into weed science and to the preparation of new students who will take us into the future. The points are not given in a particular order.

(1) There is an urgent need to effectively extend the life of current commercial herbicides by reducing selection pressures on weeds, therefore preserving the genes for susceptibility. This demands:

• Improved and innovative strategies for the use of herbicides (i.e., use of full rates, adjuvants, tank mixtures, and rotations, with a focus on early-season weed management). These strategies are already practiced in developed countries but need to include in developing countries also.
• More research devoted toward the manipulation of non-chemical means, and the optimization and integration of these methods with chemical weed control methods.
• Strategic and eco-efficient use of herbicides aimed at improving efficiency through the development and promotion of precision application techniques. The goal of such a strategy is to maximize delivery to the intended weed flora, avoiding non-target application and minimizing negative environmental impacts.
• A change in perspective and approach from short-term weed control to sustainable weed management.
• Studies devoted to assessing the impact of herbicide usage on species richness, diversity, and abundance of resistant/tolerant weed species. The response of weed populations to herbicide-exerted selection pressure, and remedial measures, should be ascertained.

(2) Strengthening research efforts to discover alternate approaches to manage weeds, for example:

• The discovery of novel active ingredients, and commercialization of new herbicide products capable of controlling both susceptible populations and resistant weed biotypes.
• Identifying new morpho-physiological or biochemical traits conferring multiple herbicide tolerance, and the incorporation of such stacked traits into various crop types, to continue to benefit from existing chemical products, keeping in mind the pragmatic and judicious use of these herbicide-resistant crop traits to reduce environmental impacts and prevent selection of rare herbicide-resistance alleles in natural weed populations.
• More focused research on weed ecology in terms of reducing weed seed banks in the soil and use of HWSC methods.
• The development of stimulants and desiccants to manipulate germination and dormancy mechanisms of weed seeds, with the aim of reducing soil seed banks. The inherent and inducible karrikinolide response of weeds belonging to different families with contrasting dormancy status, in conjunction with variable regimes of light and temperature, should be investigated.
• The development and commercialization of state-of-the-art technologies [e.g., bio-control methods, RNAi (RNA interference), etc.].

(3) Research efforts are needed to refine IWM principles for various cropping systems and agro-ecological regions. More holistic research and development programs are needed to manage weeds over multiple seasons. The impact of long-term fertilization practices on weed species composition, abundance, diversity, and functional traits should be worked out across conventional and conservation tillage systems, while considering spatial heterogeneity of the landscape. This approach will help predict future weed problems, so that weed management approaches can be modeled in anticipation.

(4) Weeds can be exploited as a source of valuable genetic materials for crop breeding programs – breeding for abiotic stresses (salinity, drought, submergence, and temperature stress). Genes encoding functional substances should be cloned and introduced into crops to develop stress-tolerant ideotypes.

(5) Studies on the mechanisms of herbicide resistance have revealed that plants can evolve a fascinating biological arsenal as a defense. Unraveling the complexities in metabolic-based resistance is a challenge that has the potential to cause a paradigm shift in our understanding and management of resistant weeds. Basic and fundamental research on the mechanistic and genetic basis of resistance must contribute to discovering the missing links in the evolutionary path to herbicide-resistance at genotypic, population, and ecosystem levels. Future research must focus on questions about genetic variations versus novel resistance mutations, fitness benefits, and costs under herbicide selection, as well as the links between metabolic resistance and general detoxification pathways involved in stress-response dynamics.

(6) It has been suggested that the genotypic variation among crop cultivars responsible for weed tolerance can be exploited as an integral component of IWM programs (Mahajan and Chauhan, 2013). Breeding weed-competitive (high early vigor) and allelopathic crops that suppress/kill weeds can help toward ecological weed management. Understanding the genetics of a crop’s allelopathic activity remains a germane issue to be researched (Bunce and Ziska, 2000; Fuhrer, 2003). Coordinated breeding programs focusing on the location of genes involved in the production of allelochemicals, control of the allelopathic activity, and mapping the populations between allelopathic and non-allelopathic accessions can be crucial in this regard (Fragasso et al., 2013). Identification of crop cultivars with strong allelopathic potential can contribute directly to weed suppression by their inclusion in crop rotation, or their use in breeding programs to incorporate allelopathic traits into future genotypes, making them more able to compete with and suppress weeds (Aslam et al., 2017).

(7) Real-time integration of knowledge on agronomic weeds (history, biology, ecology, and control methods) with advancements in computer science and engineering could help secure environmental protection, agro-ecosystem sustainability, growers’ profit, and public health (Singh et al., 2011). Development of efficient remote sensing and guidance systems capable of combining recognition (detection through field scouting) and application (spraying, cultivation, and mowing) modules into a single real-time platform is a critical area of research for decision support systems and site-specific weed management (Young, 2012).

(8) More sophisticated computer-based simulation models capable of integrating available information and predicting competition and population dynamics are needed for a better understanding of weed-crop relationships across a range of weed management spectrums (Renton and Chauhan, 2017). Against the backdrop of climate change, predictive modeling of weed distribution, range expansion, and invasion potential has become more critical than ever and needs to be finely-tuned and concomitantly updated to predict weed responses (Clements et al., 2014). Besides prediction modeling, models for decision support are also needed to explain the likely outcome of different management interventions, associated costs, risks involved, and potential benefits.

(9) Assessing the fate and behavior of applied herbicides in various cropping systems remains a neglected area of research in developing countries. We understand that information on the fate and transport of herbicides in the environment is over-whelmed in the literature; however, most of this information is from the studies conducted in advanced countries. Far-reaching research is needed to quantify persistence and mobility of commonly used herbicides in the rice-wheat cropping system, and to explore their environmental fate. Residual herbicide analysis can be helpful in predicting the nature and level of contamination in the litho and hydrosphere, and the implications for the biosphere as a whole. Understanding these processes can yield information about introduction and degradation pathways into the environment, facilitating risk assessment (Francaviglia and Capri, 2000) that can serve as a basis for modeling (Nhung et al., 2009). Moreover, the impact of these persisted chemicals on succeeding crops also needs to be investigated.

(10) Focused research is needed to unravel mechanisms conducive to the success of alien invasive weeds and identify vulnerabilities, to inform monitoring, early detection and warning systems, assist development of regional and global databases, strengthen quarantine and management systems, assess ecological and economic impacts, and improve public awareness.

(11) The impact of climate change on crop-weed competitive outcomes needs to be studied, considering all possible combinations of plant-weed carbon fixation pathways, C_3_ crops and C_3_ weeds, C_4_ crops and C_4_ weeds, C_3_ crops and C_4_ weeds, and C_4_ crops and C_3_ weeds. To what extent, the so called “CO_2_ fertilization” could compensate for other negative effects of climate change on crop-weed competition remains elusive. Competitive outcomes in managed and natural ecosystems pertaining to agricultural and invasive weeds need to be carefully studied considering the projected increase in CO_2_ concentration, in conjunction with associated variations in other climatic variables such as temperature, rainfall, and drought. Against the backdrop of climate change, the production and concentration of secondary metabolites associated with allelopathic activity, geographic distribution of invasive weeds, and toxicity of poisonous weeds need to be explicitly assessed. Development and promotion of adaptive mechanisms and innovative practices to cope with weed problems under climate change is needed for sustainable crop production. A judicious analysis of their effectiveness, economic and ecological costs, and time span required is also essential in this regard.

(12) Apprehension about herbicide-resistant crops, such as negative impacts on biodiversity, gene transfer between wild relatives (particularly in the centers of crop origin), development of super weeds, and health issues, warrant the need for educational and awareness activities in collaboration with public groups, stakeholders, and policy makers, to foster the adoption process (particularly in Asia). The seed biotech industry should devise safe mechanisms of transgenic development to avoid introgression of resistant genes to related weeds.

(13) In order to harness the benefits of weed science for sustainable crop production, capacity building of scientists, teaching and training staff, extension personnel, and agri-graduates needs to be alleviated. Networking and collaboration of experts, knowledge sharing, and technology transfer from developed countries could benefit the overall weed science discipline in the developing world. Increased cooperation between complementary research groups of weed scientists (working in areas of IWM, herbicide efficacy, herbicide resistance, invasive plant management, ecological weed management, genetics, molecular biology, morphology and physiology of weedy traits, and ecosystem restoration) will be a step toward rediscovering and answering critical research questions, thereby enabling the weed science discipline to better respond to modern day vegetation management challenges and issues.

(14) Weed science educators/instructors should devise more experiential learning activities (fact-based learning processes that integrate tangible experiences, insightful observations, abstract conceptualization, and vigorous experimentation; reviewed by Atherton, 2002), and incorporate the same into their courses in order to promote understanding of the subject matter and concept retention (Gallagher et al., 2007).

## Conclusion

Weed science as an applied and integrative scientific discipline combines basic and applied sciences to better understand and manage weeds. Proper weed management promises food security via enhanced productivity and profitability, while safeguarding the natural resource base. Successful identification and alleviation of weed threat is one approach to enhance yield and abridge yield gaps. Weed scientists have a daunting job to deal with a plethora of problems that, although relevant, remain unexamined. Modern day weed management issues and challenges urgently demand weed scientists look beyond the herbicide efficacy/fate box and probe into basic and applied research pertaining to complex vegetation management in both natural and managed ecosystems that are currently tackled by plant physiologists, molecular biologists, and invasion ecologists.

To overcome various technical challenges, recent decades have witnessed significant progress in the form of site-specific weed management systems, herbicide-resistant transgenic crops, drones to monitor weed population dynamics, omics, novel herbicides, molecular biology tools, nanoherbicides, and simulation and decision support modeling. The human dimension is somewhat more difficult, and weed science has to grapple with issues such as farmers’ failure to appreciate the extent of weed menace, especially where the damage and losses are not apparent. Assessment of the environmental impact of weed management practices has formed a new and a relevant area of research in weed science. Against the backdrop of precision agriculture, advancements in the field of engineering and computer sciences can help quickly identify and control weeds with precise recognition and application modules. For weed science to thrive and respond to future weed problems, greater global collaboration will be required between this discipline and biological science, computer science, engineering, economics, and sociology. Channelizing and harnessing interdisciplinary collaboration and training of weed scientists, coupled with information exchange, could help solve complex challenges with more diverse and versatile approaches, and achieve greater consensus – so avoiding uncertainties and critiques.

Weed scientists should respond to their critics by taking part in reflection, introspection, and debate. In particular, steps should be taken to avoid a preponderance of repetitive and descriptive studies on herbicides. We believe that a paradigm shift in weed science will begin with a paradigm shift in the “way weed scientists think and pose critical research questions and hypotheses.” As weed scientists, we should acknowledge a general lack of diversity that currently exists in weed management programs. We can then enrich our tool kit by using exciting novel tools from other disciplines, and should seek the opinion of intellectuals from diverse scientific backgrounds. Future weed science is expected to be a perfect blend of different disciplines-all contributing to a unifying goal of sustainable weed management.

It is now well-known that increased global trade is also resulting in exotic weed spread, potentially creating alarming new situations in the wake of climate change. Therefore, advanced knowledge in weed science will be required to provide new tools for handling such complex emerging problems for weed management in the 21st century. As a direct consequence of this scenario, weed scientists will need to revisit the concept and tactics of IWM, since its non-chemical components are currently being given less priority by both public research institutes and the farming community, with continued reliance on synthetic chemicals. We urge that innovative and diverse teaching practices should be developed to prepare weed science graduates for the anticipated challenges of future agriculture. Currently, weed science research, education, and extension is lagging behind the priority needs of weed management in natural, agricultural and urban landscapes, and the situation is expected to worsen with climate change. Increased resource mobilization and funding could prove beneficial in this regard. The number of positions devoted to weed science research, teaching, and extension needs to be increased, especially in areas where acute shortages are evident (such as natural ecosystems and non-cropland weeds, and invasive plant management). In a similar way to other plant protection disciplines (plant pathology and agricultural entomology), weed science should also be promoted to be a major department of all agricultural universities, offering innovative graduate- and post-graduate degree programs.

## Author Contributions

BC, AM, and GM developed the initial concept and outline and they took lead in expanding the content. FA, PJ, and SF contributed and edited the manuscript.

## Conflict of Interest Statement

The authors declare that the research was conducted in the absence of any commercial or financial relationships that could be construed as a potential conflict of interest.
